# A Smart Toy to Enhance the Decision-Making Process at Children’s Psychomotor Delay Screenings: A Pilot Study

**DOI:** 10.2196/jmir.7533

**Published:** 2017-05-19

**Authors:** María Angeles Gutiérrez García, María Luisa Martín Ruiz, Diego Rivera, Laura Vadillo, Miguel Angel Valero Duboy

**Affiliations:** ^1^ Universidad Autónoma de Madrid Departamento de Didáctica y Teoría de la Educación, Facultad de Formación de Profesorado y Educación Madrid Spain; ^2^ Grupo de Investigación Tecnologías para la sociedad de la información y el conocimiento (T>SIC) Departamento de Ingeniería Telemática y Electrónica Escuela Técnica Superior de Ingeniería y Sistemas de Telecomunicación, Universidad Politécnica de Madrid Madrid Spain; ^3^ Universidad de Alcalá de Henares Departamento de Automática, Escuela Politécnica Superior Madrid Spain

**Keywords:** research instruments, questionnaires and tools, Information retrieval, Internet of things, clinical information and decision making, Web-based and mobile health interventions, developmental delays, smart toys

## Abstract

**Background:**

EDUCERE (“Ubiquitous Detection Ecosystem to Care and Early Stimulation for Children with Developmental Disorders”) is an ecosystem for ubiquitous detection, care, and early stimulation of children with developmental disorders. The objectives of this Spanish government-funded research and development project are to investigate, develop, and evaluate innovative solutions to detect changes in psychomotor development through the natural interaction of children with toys and everyday objects, and perform stimulation and early attention activities in real environments such as home and school. Thirty multidisciplinary professionals and three nursery schools worked in the EDUCERE project between 2014 and 2017 and they obtained satisfactory results. Related to EDUCERE, we found studies based on providing networks of connected smart objects and the interaction between toys and social networks.

**Objective:**

This research includes the design, implementation, and validation of an EDUCERE smart toy aimed to automatically detect delays in psychomotor development. The results from initial tests led to enhancing the effectiveness of the original design and deployment. The smart toy, based on stackable cubes, has a data collector module and a smart system for detection of developmental delays, called the EDUCERE developmental delay screening system (DDSS).

**Methods:**

The pilot study involved 65 toddlers aged between 23 and 37 months (mean=29.02, SD 3.81) who built a tower with five stackable cubes, designed by following the EDUCERE smart toy model. As toddlers made the tower, sensors in the cubes sent data to a collector module through a wireless connection. All trials were video-recorded for further analysis by child development experts. After watching the videos, experts scored the performance of the trials to compare and fine-tune the interpretation of the data automatically gathered by the toy-embedded sensors.

**Results:**

Judges were highly reliable in an interrater agreement analysis (intraclass correlation 0.961, 95% CI 0.937-0.967), suggesting that the process was successful to separate different levels of performance. A factor analysis of collected data showed that three factors, trembling, speed, and accuracy, accounted for 76.79% of the total variance, but only two of them were predictors of performance in a regression analysis: accuracy (*P*=.001) and speed (*P*=.002). The other factor, trembling (*P*=.79), did not have a significant effect on this dependent variable.

**Conclusions:**

The EDUCERE DDSS is ready to use the regression equation obtained for the dependent variable “performance” as an algorithm for the automatic detection of psychomotor developmental delays. The results of the factor analysis are valuable to simplify the design of the smart toy by taking into account only the significant variables in the collector module. The fine-tuning of the toy process module will be carried out by following the specifications resulting from the analysis of the data to improve the efficiency and effectiveness of the product.

## Introduction

In early childhood, children learn by playing in natural settings such as the playground, home, or kindergarten. By playing, children develop various cognitive, perceptual, motor, linguistic, and communicative skills. When a child plays alone, he or she interacts with objects, usually toys, and performs various movements, such as picking or throwing objects, placing them in a row, or stacking them on top of one another. Not all these movements are simple; for example, building a tower with stackable cubes can be difficult for children between ages 2 and 3 years, especially if the cubes are small and the tower is high. Standardized developmental tests include items that ask children to make a tower by stacking cubes [1]. At 24 months of age, children must be able to stack at least five cubes. If a child does not succeed, he or she will be below the standard 24-month-old score, which may indicate some kind of delay in psychomotor development, the seriousness of which depends on the number of cubes the child can stack. Of course, a child development test is composed of several items that measure not only psychomotor development, but also language development, for example, through the understanding of instructions.

The research performed in the EDUCERE (Ecosistema de Detección Ubicua, atenCión y Estimulación tempRana para niños con trastornos del dEsarrollo; “Ubiquitous Detection Ecosystem to Care and Early Stimulation for Children with Developmental Disorders”) project focuses on psychomotor development and analyzes how toddlers build a tower of cubes by electronically recording all significant movements performed by the children. The tower of five stacking cubes is an example of the psychomotor behavior of toddlers aged between 2 and 3 years. The aim is to determine if children are capable of building the tower and to analyze how they do it in order to detect minimal delays in development, which may lead to preventive monitoring of some children or, when appropriate, to the implementation of an early attention program.

Ubiquitous computing and ambient intelligence could support innovative application domains, such as the detection of motor impairments within the home environment [2]. Hence, the embedding of different kinds of sensors into everyday toys will allow the collection of systematic information processes and actions in order to make an early detection of potential problems that may affect development in the field of mobility. Furthermore, the detection of a potential motor problem paves the way to the utilization of this technology for early attention to children through educational activities that can mitigate possible additional effects in the future.

Further to this contextualization, the goal of the EDUCERE project [3], including the cube-based smart toys design presented in Rivera et al [4], is to build a developmental delay screening system (DDSS) at home or school that can record children’s behavior and skills in order to detect early psychomotor developmental problems and promote stimulation activities. A multidisciplinary group of 30 researchers specialized in disciplines of childhood development (educators, psychologists, physiotherapists) and computer science engineers defined the smart toys model, the set of detectable measures, the embeddable sensors kits, and useful numeric information. The first toy selected by the working team was a set of cubes and the defined activity was to build a tower of cubes [4]. The aim of the DDSS is to provide authorized professionals with sufficient reliable information about the activity performed by a child. Smart toys help professionals by giving information about the following parameters [4]: (1) motion pattern while the child is moving a cube, including time of activity, acceleration, speed, and shaking data, and (2) tower status, including knowledge about how the children made the cube tower, how long it took, and how accurate was the alignment of cubes in the tower.

Early detection of developmental problems is a critical matter to assure the wellness of children [5]. Nowadays, most experts use different activities to evaluate the evolution of child development and motor skills, and many of these activities involve the manipulation of toys and other objects [6]. In fact, there are developmental scales, such as Merrill-Palmer [1] or Bayley [7] scales, which use specific toys and activities done by the child and are employed to identify possible delays or difficulties.

There are many childhood disorders, such as autism spectrum disorders, that can be detected by using information from the child’s movements in certain activities [8-10]. Therefore, using sensors to obtain this kind of information is a logical step toward a more accurate detection process [11]. For instance, Marschollek et al [12] showed a classification of sensors to be used for these tasks, although oriented to wearable devices. In addition, unobtrusive wearable devices such as wristbands help to detect and measure movements [13]. Moreover, Taffoni et al [14] described how wearable sensors can measure children’s movement when stacking a pile of cubes.

These approaches are partially intrusive because children have to use special wearable devices that are not part of their everyday routines. The goal of the EDUCERE project is to embed the measurement tools in ordinary objects, extending the Internet of things (IoT) paradigm to an “Internet of toys” experience by creating smart toys based on everyday objects equipped with low-cost sensors. This allows the acquisition of accurate information without interfering in children’s daily activities.

This approach to the IoT using toys as smart objects is not exclusive for the purposes presented in this paper. For instance, Wang et al [15] explored the relationship of the IoT and toys (Internet of toys) in terms of interaction between toys and social networks. In addition, the Disney Research laboratories worked in the Internet of toys through the European CALIPSO project [16], which the main goal was to provide networks of connected smart objects, but their main efforts were on the design of low-consumption and low-latency communication protocols between the objects [17].

### Materials

#### Smart Toys Development

The EDUCERE project aims to create a whole ecosystem of compatible smart toys that provide information that helps child development professionals to detect potential developmental problems. The first toys created for that purpose were a set of cubes that can be stacked to build a tower [4], but other compatible toys, such as pegboards, rattles, or balls, were designed. As a design consideration, we established that the cube must be safe for child interaction. Hence, the cubes cannot open while the toddler plays with them to avoid smaller pieces inside the block from causing harm to the toddler.

All toys include the ATMEL ATmega328p microcontroller by Atmel Corporation (San Jose, CA, USA). This controller is integrated with Arduino boards and is compatible with them, offering an easy and fast prototyping platform to develop the toy software through the Arduino integrated drive electronics. Each toy includes the sensors that add the needed functionality in each case. Every toy uses a NRF24 radio frequency adapter for communications with a data collector device. The NRF24L01+ by Nordic Semiconductor ASA (Oslo, Norway) is an ATmega328p-compatible 2.4 GHz radio frequency transmitter/receiver chosen because of its size, low cost, and low consumption.

Specifically, the stackable cubes consist of the preceding components and:

1. A 3.7 lithium-ion polymer battery. The main limitation for the battery was the maximum size of the cubes (2.5 cm per side), so 150 milliamp hour was the maximum capacity to be fitted in the available space. In addition, a protection circuit was included to ensure that the battery never offers less than 3.2 volts.
2. A set of 12 (two per cube face) light-dependent resistor sensors from Silonex Inc (Montreal, QC, Canada). These sensors allow for determining which face of the cube is covered at each moment.
3. A MPU-9150 InvenSense by Sunnyvale (San Jose, CA, USA) with a three-axis accelerometer and gyroscope. This sensor provides speed, acceleration, and shaking level values for each cube movement.
4. A tilt-based switch that enables a sleep mode to decrease power consumption.
5. Three light-emitting diodes (LEDs) and a buzzer for a visual and auditory user interface.

**Figure 1:**
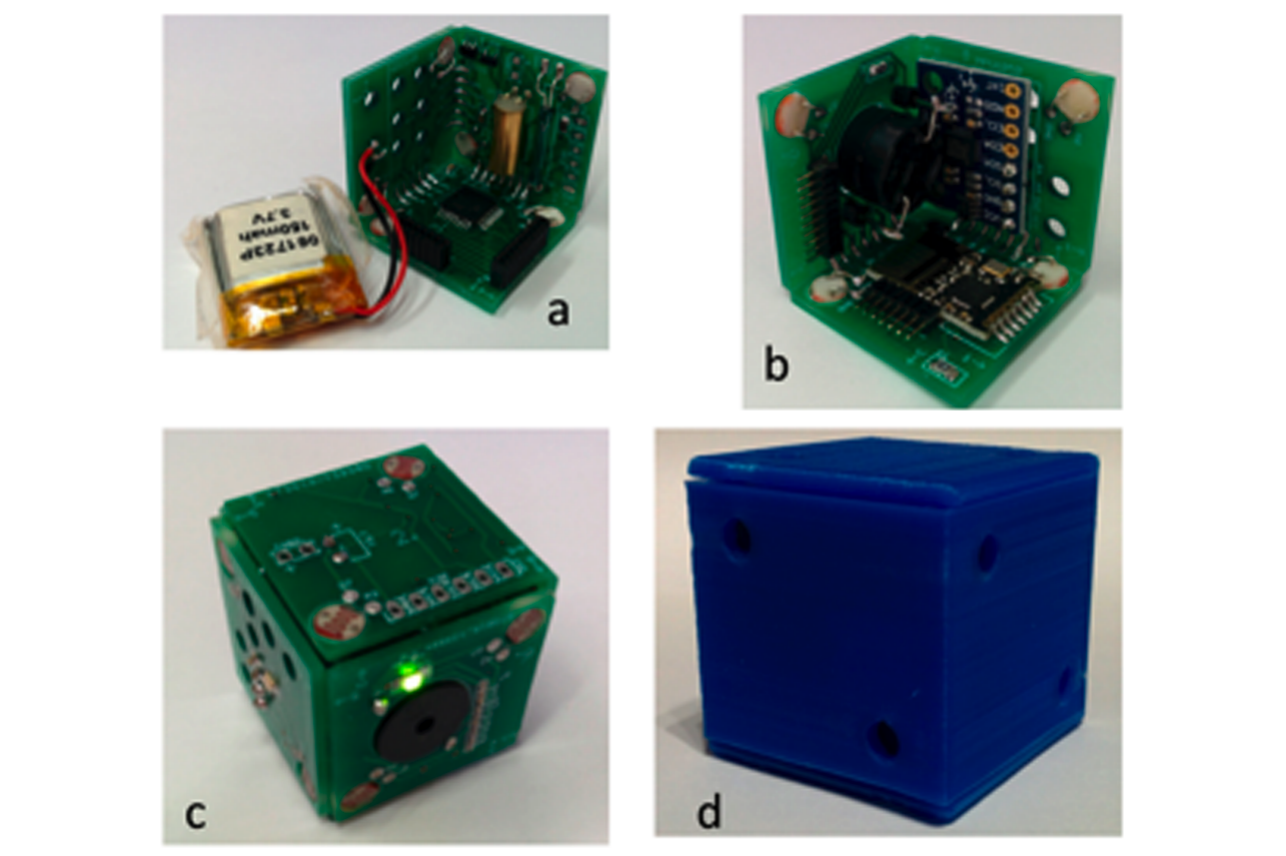
The cube printed circuit board construction (a-c) and the 3D printed external case (d).

The very strict size constraint in cube size (no more than 2.5 cm for each face) required designing a specific printed circuit board (PCB) shaped as a cube itself. The PCB is divided into six square faces and each face contains part of the printed circuit. Figure 1 shows how the faces are welded together at 90 degrees creating two pieces that are assembled together as a cube. Finally, a plastic 3D printed case was designed to cover the circuit board. A prototype set of 10 cubes was built for the laboratory experiments initially, but more cubes were assembled allowing more experiments to be performed in parallel in different schools.

Cubes transmit the gathered data to a collector module that gives format and stores and encrypts the information. The collector module has been deployed in a Raspberry Pi board with a NRF24L01+ adapter that allows it to connect by itself to the toys. The collector provides a RESTful application program interface through a Wi-Fi access point that allows for managing the experiments and the data files obtained in these experiments using a native app in a tablet or a phone. The data files are cyphered using Advanced Encryption Standard (AES) on Cipher Block Chaining (CBC) mode with a one-use 128-bit key. The key is randomly generated for each experiment and is also encrypted with the RSA DDSS public key to ensure the information is only accessible in a secure Web server.

#### The EDUCERE Developmental Delay Screening System

**Figure 2:**
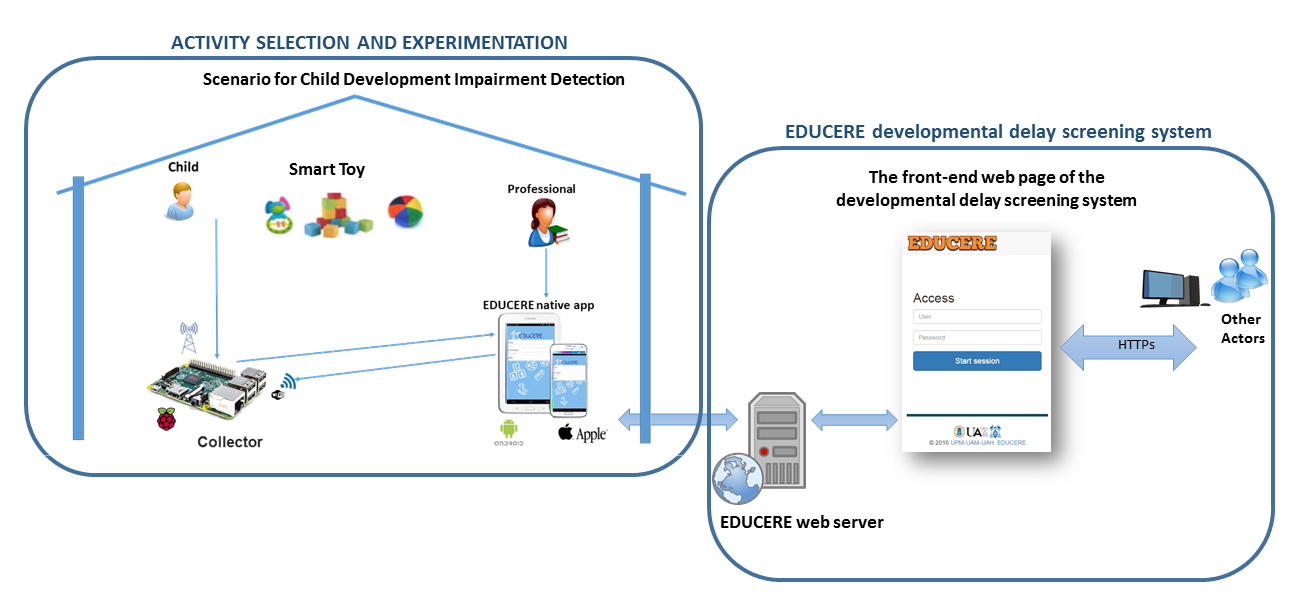
Components of the general architecture in EDUCERE developmental delay screening system.

Figure 2 shows the general component architecture of the EDUCERE DDSS. The left side (activity selection and experimentation) shows the necessary elements for the experiment: the child playing with the toy, the smart toy, a professional to assist the child, a Raspberry Pi (used as collector to obtain and save the received data from the smart toy with the rest of experiment information), and a tablet with a mobile app to interact with the collector (for starting, finalizing, repeating, and storing the experiments). The right part (EDUCERE DDSS) shows the components for registry, consultation, and modification of information about children, professionals, and experiments. Professionals can also perform the analysis of the results obtained by the children with the smart toys interaction.

The DDSS of EDUCERE contains all administrative tasks to securely register children, professionals, and activities used during the experimentation scenario. The registry process is done in two steps:

1. Before beginning the process of experimentation with the explicit smart toy, a user with an administrative role will access the EDUCERE system to store the following information: the specific smart toy for the experimentation, the activities that can be performed with that smart toy, and the professional who will carry out the experiment.
2. After that, the administrative user reports to the professional so that they can begin the experimentation process. The professional must be authenticated in the EDUCERE DDSS and enter the information requested for each of the children who will be performing the experiment.

The person with the professional role must upload information about the children who will carry out the experiment with the smart toy. The professional enters name, date of birth, gender, the name of the professional who will carry out the experiment, and the name of the center where this experiment will be done. The professional must also record in EDUCERE the information about the activities that the child could do with the smart toy.

**Figure 3:**
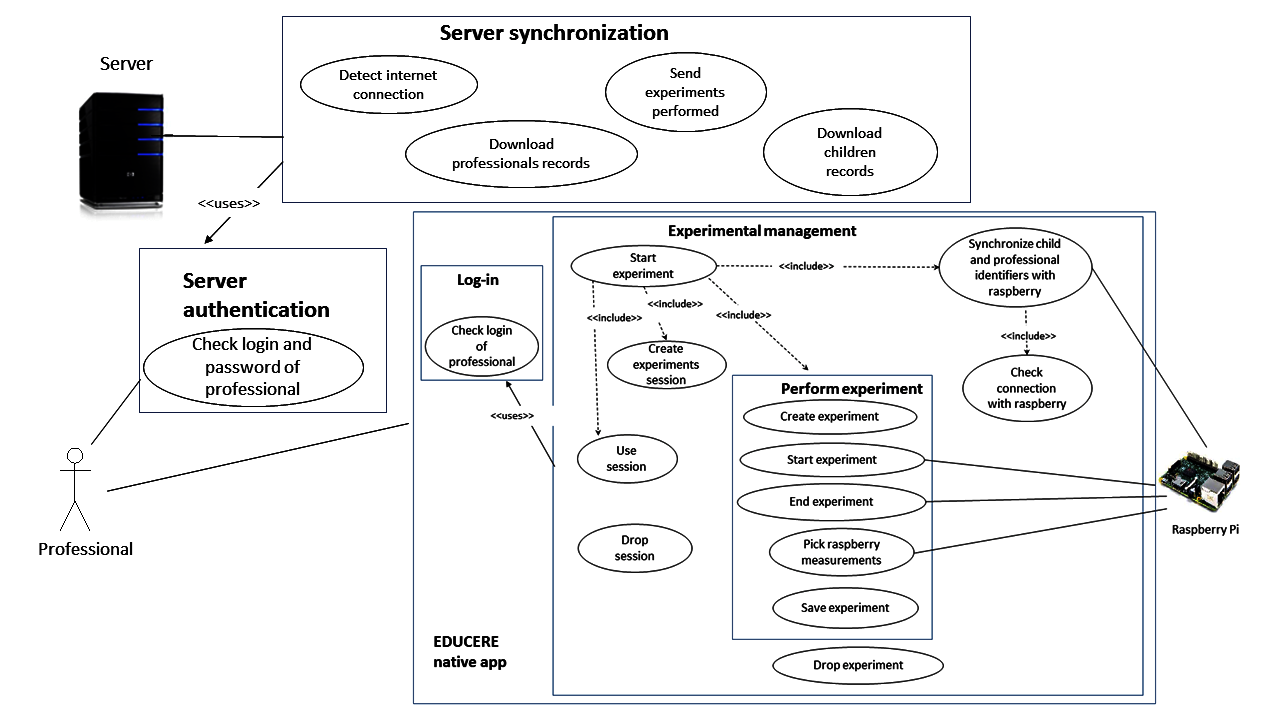
EDUCERE mobile app use case diagram.

Several children participate at each screening session. In addition, the experimentation session can last for several days with the whole group. Figure 3 shows the use case diagram with the main functionality of the mobile app developed for the tablet.

Use cases of the mobile app consist of three main stages:

1. Experimental management: includes all use cases related to the experimentation process. Users log in to the mobile app to access, such as a known professional. In this case, only the log-in of the professional is required. Before starting experiments, the mobile app (previously connected to the EDUCERE Wi-Fi generated by the Raspberry Pi collector) synchronizes professionals and children with the collector. To start experiments, it is required to create or use an experiment session because all experiments will belong to a concrete session. The professional could also drop a session when the session is over. Once the professional has selected a session, the professional should configure the experiment, choosing a registered child and the cube activity. The experiment starts when the professional presses the “start experiment” button. The experiment stops when he or she presses the “stop experiment” button. Next, the professional can refuse it or store it. If the professional chooses to store it, the collector sends all data collected during the experiment in an encrypted file form. This file is saved in the internal storage of the mobile or tablet.
2. Server synchronization: there are two ways of synchronization between the mobile app and the EDUCERE DDSS server. Before the experiment starts, the mobile app connects with the EDUCERE DDSS server to download information about registered children and professionals. The mobile app uses this information to synchronize the professionals and children with the collector. After performing experiments, the mobile app connects with the EDUCERE DDSS server to upload experiment files with the collected measurements to the server.
3. Server authentication: to synchronize the mobile app with the EDUCERE DDSS server, the professional must be authenticated in the app (submitting log-in and password) to access to the server. This log-in and password are sent to the server to start synchronization.

The mobile app has been implemented in HTML5-Javascript for cross-platform development, using jQuery and the responsive Web app development framework Bootstrap. Through Cordova, a well-known open source mobile development framework, the wrapper is generated to run the app on different mobile platforms, such as Android or iOS. The result is a hybrid app executed via Web views. Cordova also provides a series of plug-ins to access the functionalities of the mobile device, such as internal storage, connection detection, and vibration, all of which are necessary for the development of the mobile app.

**Figure 4:**
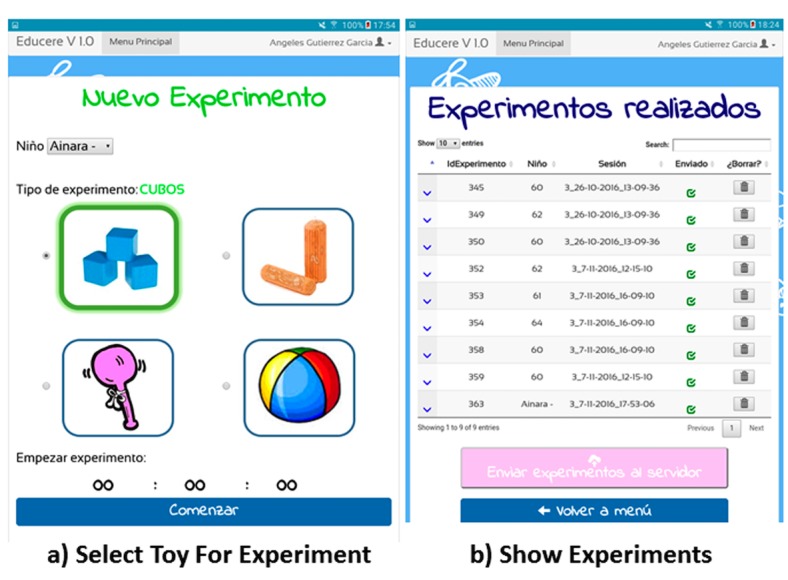
EDUCERE mobile app interfaces (in Spanish). The professional can select the toy for experiment (a) and show data from experiments performed (b).

Figure 4 shows an example of two interfaces of the mobile EDUCERE app. Part A displays the interface to start a concrete experiment with a child by using one of the smart toys developed. Through this user interface, professionals can choose the child and the type of experiment. The “start experiment” button sends the signal “start” to the collector to start storing data from the experiment. Part B contains information of all performed experiments by the professional. This screen includes the button “send all experiments to the server” to upload json experiment files to the EDUCERE DDSS.

**Figure 5:**
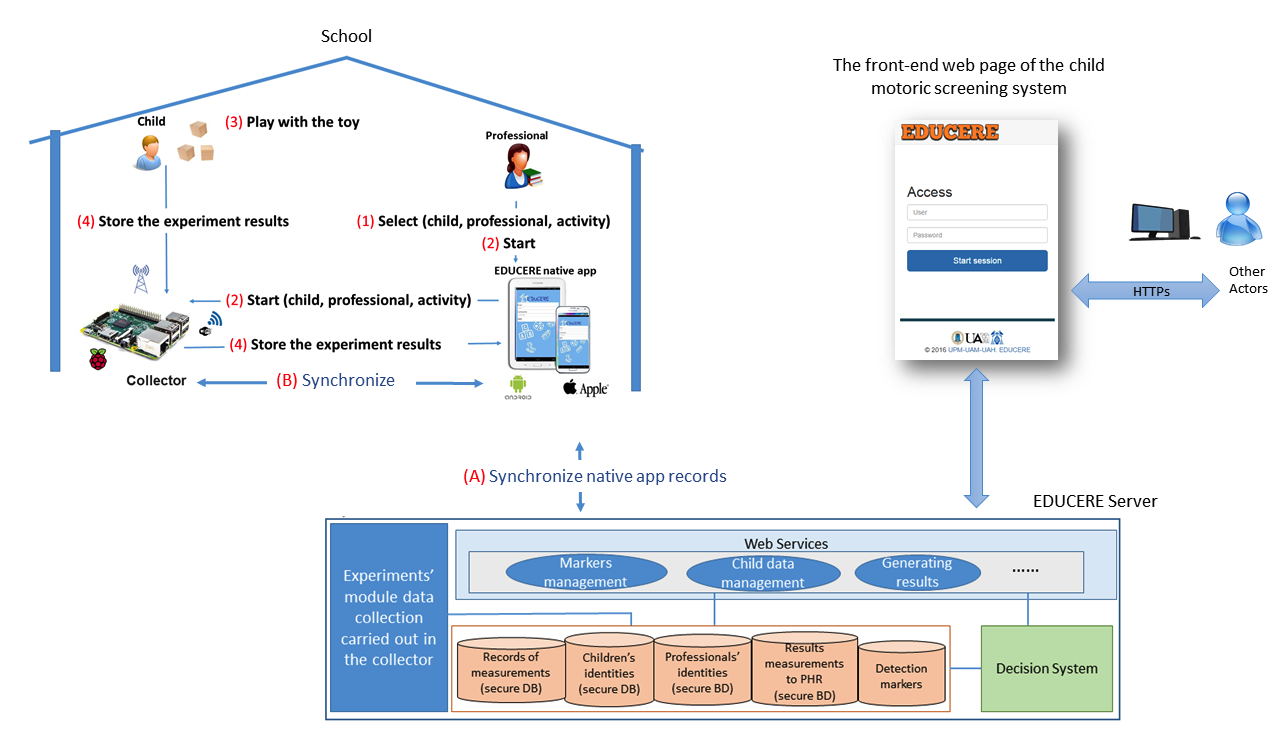
Steps for interaction with the EDUCERE developmental delay screening system.

During the experimentation scenario, the records of the activities performed by the child with the smart toy are collected and sent to the EDUCERE DDSS to be persistently stored (see Figure 5). In addition, the EDUCERE DDSS checks the results obtained by each child with the smart toy. Thus, the information stored in the EDUCERE system can provide professionals with useful information about early detection of a child’s motor difficulties.

Both in the school or home scenario, the child registered in the EDUCERE DDSS performs the activity indicated by the professional with the specific smart toy (rattles, balls, and cube towers). In this task, the professional assesses the child during the experimentation process and takes responsibility for the experiment. He/she uses a tablet to perform the process of starting the experiment, finalizing the experiment, and storing it. This is the way in which communication occurs between the professional and the collector, who manages the activity to be performed with the smart toy.

As Figure 5 outlines, the professional should take the following steps to guide the child in the interaction process with the game:

1. When opening the app, the displayed home screen helps to identify the professional who will perform the experiment. Once the professional provides identification, he or she selects the child who will perform the experiment. After that, the professional chooses the cube tower activity, which is the activity to be performed in which the child plays with a set of cubes.
2. Start experiment. At this point, it is necessary to synchronize the collector with the identifiers that are in the tablet corresponding to the child and professional (steps A and B in Figure 5). Then, the professional chooses a session already created or he/she starts with the creation of a new session. This distinction is necessary because the experimentation process with a set of children can be done over several days and it may be necessary that all the experiments belong to the same session, although they take place on different dates.
3. After that, the child begins interacting with the toy and the information generated by the toy’s sensors is stored in the collector. When the child completes the activity with the smart toy, the professional indicates on the tablet that the experiment has ended.
4. At this step, the experiment data in the collector are transmitted to the tablet and securely stored.

Finally, when the mobile app detects a known Wi-Fi, it connects to it to synchronize with the EDUCERE DDSS server (always prevails over all Wi-Fi raspberry, to which you must connect to start the experiment). The tablet connects to a specific Web service in the server to synchronize and transfer information between the experiments stored in the tablet and the EDUCERE system databases on the server (step A in Figure 5). The EDUCERE databases contain information that identifies registered professionals in the system and the children who have been discharged by each professional, and data from all experiments uploaded by the mobile app.

## Methods

### Participants

A total of 65 toddlers (32 boys and 33 girls) from a public nursery school aged between 23 and 37 months (mean 29.02, SD 3.81 months) took part in a pilot trial in which they had to build a tower with five stackable cubes. The professional did not have clinical information about the toddlers who participated in the experiment. Parents signed an informed consent sent by EDUCERE project.

### Apparatus and Materials

**Figure 6:**
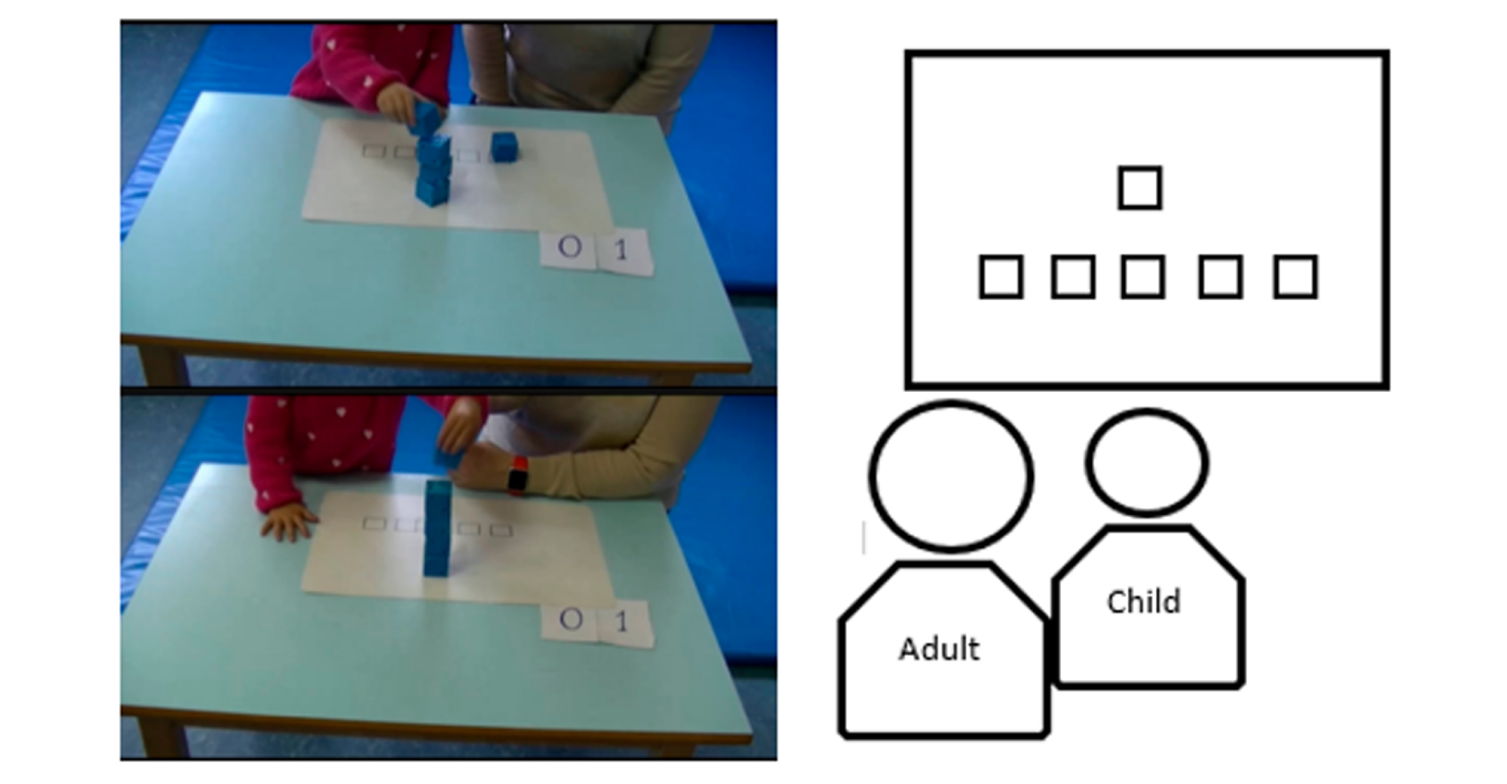
Experimental scenario. Initially the five cubes are placed in a row on the template and the child is to build a tower with the cubes on the square in front.

The toddlers were given five EDUCERE stackable cubes placed in a row on a template (Figure 6). Thus, the initial positions of the smart cubes and the place to build the tower were marked to align the trial.

The observer sat to the left of the child who was in front of the table in the middle. During the experiment, the experimenter needed the following elements: (1) a collector module (described in Smart Toys Development subsection) to format, store, encrypt, and transmit information obtained from the cubes; (2) a tablet with a mobile app (as shown Figure 4) to interact with the collector for starting, finalizing, repeating, and storing the experiments; and (3) a video camera to record all trials for later viewing.

### Procedure

The set up of the trials was fixed on the tables used by the toddlers. The professional made the tower with the stackable cubes in the marked position while saying, “Look, I’m going to make a tower with these cubes right here” (see Multimedia Appendix 1, Table S1). After a few seconds and making sure that the toddler looked at the tower, the professional put the cubes back in their initial positions. Then the adult asked the child to make the tower (“Now I would like you to make a tower just like I did”) and waited for the child to make the tower. If the child dropped the pieces, the professional suggested putting them back on the tower. Children could make as many attempts as they wanted and all data were recorded but, once the video was visualized, the first attempt was selected for analysis. Finally, all toddlers received a sticker as a reward.

Four experts in child psychomotor development (one developmental psychologist, one physiotherapist, and two educators) viewed the recordings and selected an analyzable fragment and scored each child trial on a scale of 1 to 10 according to their performance. The experts had a meeting prior to viewing the videos in order to agree on assessment criteria (see Multimedia Appendix 1, Table S2).

Each expert randomly viewed half of the videos and two experts individually rated each video. In this way, two experts scored each child’s performance. The experts made their assessments without having contact with one another to ensure their independence of judgment. Because the experts came from different professional backgrounds, it was expected that their assessments would not be identical, despite having reached agreement on the criteria. Multimedia Appendix 1 (Table S3) shows the professional profiles of the four experts.

The selected video fragments had their corresponding data recorded by the collector module. These data were included, together with the expert scores, in the statistical analysis.

### Design

The set of variables for each experiment were measured by using the sensors included in the toys (see Smart Toys Development subsection). The toy processes these variables starting from the values obtained from the sensors and stores them for each movement. The variables are the maximum acceleration during the movement, the maximum and mean speed, the time at which the maximum speed is reached within the movement, the time the movement took, and the number of shakings detected. The shakings are calculated from the graph of instantaneous accelerations by considering a shaking as data between two minimum values. There were four levels of shakings. In order to determine the level of shaking, the number of samples that fit the “mound” in the accelerations graph was taken into account. Using this classification scheme, the first level represented the smaller shakings (ie, the shorter “mounds”, where only one sample from the sensor was received before and after the maximum acceleration value) and the fourth level represented the bigger shakings (where four or more samples were received). This measurement and classification has been explained in more detail previously [4].

Once all the experiments were performed, the stored per-movement data were summarized in a per-experiment data file. The variables used in the analysis were these summarized values, including the performance scores determined by experts, which are detailed in Table 1.

**Table 1:** Summary of variables used in the analysis.

Variable name	Meaning	Dimensions/Range	How it is calculated?
Performance scores	Scores of children performing the activity	1-10 (10 being the best possible score)	It is assigned by experts while reviewing the experiment
Number of movements	Total number of movements made with all the cubes during an experiment	1-n (ideally five, one movement per cube)	A movement is any period of time in which the cube accelerometer sensor gives (after velocity calculation) a value high enough to determine the cube is moving (see [4] for a detailed explanation of the calculations)
Mean time of movement	Mean of the duration of each movement during an experiment	Milliseconds (msec)	The period of each movement is detected and stored and then the mean value of all these time values is calculated
Mean speed of movement	Mean of all the mean speed values measured during an experiment in meters per second.	Meters per second (m/s)	The speed values during a movement are calculated by integrating the values obtained by the cube accelerometer; with all the instant values within a movement, the mean speed is calculated and this value is the mean of these means for all the experiment
Mean of maximum speed	Mean of all the maximum speed values	m/s	For each speed value obtained during a movement, the maximum value is stored, then the mean of these values is calculated for the entire experiment
Highest maximum speed	The maximum value of the maximum speeds	m/s	For all the maximum values stored during an experiment, the maximum value is selected
Lowest maximum speed	The minimum value of the minimum speeds	m/s	For all the maximum values stored during an experiment, the minimum value is selected
Maximum acceleration of movement	Mean of the maximum acceleration values	m/s^2^	The accelerations are calculated directly from the values obtained in the accelerometer; the maximum value obtained for a movement is stored and, for this variable, the mean of these maximum values is calculated
Highest maximum acceleration	The maximum value of the maximum accelerations	m/s^2^	This variable represents the highest value of the maximum accelerations stored during an experiment
Lowest maximum acceleration	The minimum value of the maximum accelerations	m/s^2^	This variable represents the lowest value of the maximum accelerations stored during an experiment
Mean of shaking (level 1)^a^	Mean of the number of shaking of level 1	1-n	Given the previous definition of shaking, this variable represents the mean of the level 1 shakings measured for each movement
Mean of shaking (level 2)^a^	Mean of the number of shaking of level 2	1-n	Given the previous definition of shaking, this variable represents the mean of the level 2 shakings measured for each movement
Mean of shaking (level 3)^a^	Mean of the number of shaking of level 3	1-n	Given the previous definition of shaking, this variable represents the mean of the level 3 shakings measured for each movement
Mean of shaking (level 4)^a^	Mean of the number of shaking of level 4	1-n	Given the previous definition of shaking, this variable represents the mean of the level 4 shakings measured for each movement

^a^ The first level represents the smaller shakings (ie, the shorter “mounds”, where only one sample from the sensor is received before and after a maximum acceleration value) and the fourth level represents the bigger shakings (where four or more samples are received).

## Results

In order to analyze interrater agreement, a reliability analysis was conducted in IBM SPSS Statistics 23. We computed a intraclass correlation coefficient (ICC) analysis, following the Model ICC (1,k) in SPSS, in which 1 denotes that each participant is assessed by a different set of randomly selected raters, and k is the number of raters for every score. In this experiment, two experts out of four were randomly assigned half of the videos to rate. The other half of the videos were rated by the two remaining experts. This way, each of 65 videos had two ratings.

In the model, reliability was calculated by taking the mean of the two raters’ measurements across the 65 scores. The ICC for single measures was 0.961 (95% CI 0.937-0.976; *F*_64,64_=50.39, *P*<.001) and the ICC for mean measures was 0.980 (95% CI 0.967-0.988; *F*_64,64_=50.39, *P*<.001). The ICC of 0.980 for the mean measures indicates that 98% of the variance in the mean of these raters was “real.” The 95% CI (0.967-0.988) suggests that the process was successful to separate different levels of performance. Because the reliability among the judges’ assessment was high, we used the mean of the expert’s scores as a variable of performance in the subsequent data analysis.

A factor analysis was conducted to group similar variables into dimensions [18]. This analysis does not distinguish between independent and dependent variables, but it was useful to reduce the number of variables in the predictive regression model. We needed a predictive model to build an automatic system to support detection of developmental delays. This approach was effective for redesigning the initial prototype to be more efficient by, for example, reducing the number of sensors that focus on collecting the main relevant data.

The factor analysis showed that the first three factors together accounted for 76.784% of the total variance. Table 2 includes the rotated factor loadings, which represent both how the variables were weighted for each factor, but also the correlation between the variables and the factor. The extraction method was principal axis factoring and the rotation method was varimax with Kaisser normalization.

**Table 2:** Rotated component matrix.

Variance and variables		Component		
		1	2	3
Variance explained		31.386%	24.788%	20.616%*
**Variable, correlation estimate**				
	Number of movements	–.049	.294	–.782*
	Mean time of movement (msec)	.983*	–.048	.003
	Mean speed of movement (m/s)	.015	.840*	.199
	Mean of max speed (m/s)	–.024	.943*	–.009
	Highest maximum speed (m/s)	–.078	.723*	–.572
	Lowest maximum speed (m/s)	.035	.264	.800*
	Maximum acceleration of movement	–.083	.809*	.139
	Highest maximum acceleration	–.090	.642*	–.597
	Lowest maximum acceleration	–.044	.447	.784*
	Mean of shaking 1	.747*	–.021	–.229
	Mean of shaking 2	.896*	–.120	–.047
	Mean of shaking 3	.892*	–.024	.173
	Mean of shaking 4	.728*	.022	.225

* Strongest correlations between variables and components (factors). Those in component 1 make up “trembling” factor, those in component 2 make up “speed” factor, and those in component 3 make up “accuracy” factor.

In Table 2, the most important correlations between variables and components (factors) are marked. We assigned a name to each of these factors to represent the variables that are part of them. Based on factor loadings, we think the factors represent the following concepts:

1. Component 1 presents high correlations with the variables mean time of movement and mean of shaking (1, 2, 3, and 4). We call this factor “trembling.”
2. Component 2 indicated high correlations with the variables mean speed of movement, mean maximum speed, highest maximum speed, maximum acceleration of movement, and highest maximum acceleration. We call this factor “speed.”
3. Component 3 links high correlations with the variables number of movements, lowest maximum speed, and lowest maximum acceleration. However, the correlation with number of movements was negative, as can be observed. This means that the number of movements varies in the opposite direction to that of the other significant variables of the component and, of course, opposite the factor. We call this factor “accuracy.”

In order to design the EDUCERE automatic system for the detection of delays in toddlers’ psychomotor development using the smart stackable cubes, it was necessary to describe an algorithm that included the significant factors, or independent variables, and a dependent variable “performance.”

Two multiple regression analyses were carried out to predict (1) the value of the variable performance based on the value of the three components obtained in the factor analysis, trembling, speed, and accuracy, and (2) the value of the variable “age” based on the same three components.

Table 3 presents the model summaries for all multiple regression analyses. Table 4 presents the coefficients for all multiple regression analyses.

**Table 3:** Multiple regression analyses: model summary.

Model	Dependent	Predictor	*R*	*R*^2^	Adjusted *R*^2^	SE of the estimate
1	Performance	Accuracy, speed, trembling	.517	.267	.231	1.556
2	Age (months)	Accuracy, speed, trembling	.362	.131	.089	3.637

**Table 4:** Multiple regression analyses: coefficients.

Model		Unstandardized coefficient, B (SE)		Standardized coefficient, beta	*t*_61_		*P*	
**1**								
	(Constant)		7.662 (0.193)			39.698		<.001
	Trembling		0.050 (0.194)	0.028		0.257		.80
	Speed		–0.630 (0.194)	–0.355		–3.239		.002
	Accuracy		0.665 (0.194)	0.375		3.419		.001
**2**								
	(Constant)		29.015 (0.451)			64.315		<.001
	Trembling		–0.152 (0.455)	–0.040		–0.334		.74
	Speed		0.003 (0.455)	0.001		0.007		.99
	Accuracy		1.372 (0.455)	0.360		3.018		.004

Based on Table 3, the equation for the regression line in model 1 was: performance = 7.662 + 0.05(trembling) – 0.630(speed) + 0.665(accuracy), with the standard error of the estimate=1.556.

The coefficient for trembling (0.50) was not significantly different from zero (*P*=.80), but the coefficient was positive, which would indicate that higher trembling is related to better performance (not what we would expect). The coefficient for accuracy was positive and significantly different from zero, which means with higher accuracy there is better performance, as expected. Conversely, the coefficient for speed was significantly negative, which means that at higher speed there is worse performance.

The equation for the regression line in model 2 was: age = 29.015 – 0.152(trembling) + 0.03(speed) + 1.372(accuracy), with the standard error of the estimate=3.637.

Only the accuracy coefficient (1.372) was significant (*P*=.004). The coefficients for trembling (*P*=.74) and speed (*P*=.99) were not significantly different from zero. The accuracy coefficient was positive, which would indicate that higher accuracy was related to age (what we would expect).

## Discussion

The regression equation obtained for the variable “performance” is the algorithm that will be the basis of the automatic detection of developmental delays. In order to obtain a design as efficient as possible, the design of the smart toy must be adjusted by reducing the amount of data from the collector module taking into account the nonsignificant results obtained in the statistical analyses.

From the factor analysis, we conclude that trembling explains the greater percentage of the variance (31.38%), but considering it as a possible predictor of the performance in the regression analysis, the results show a lack of significance, so variance is unrelated to performance. This implies that the sensors that provide measurements for the variables mean time of movement and mean of shaking (1, 2, 3, and 4) have to be reconfigured to obtain only the data of interest, those that the automatic system needs to classify toddlers’ psychomotor performances. Although the prediction power of this reduced set would be slightly lower than the obtained using the original one, the reduction is probably not enough to justify maintaining the complex design of the devices.

On the other hand, speed accounts for 24.78% of the variance, but it is also negatively related to performance. For this reason, the sensors that allow the collector module to obtain measurements of the mean speed of movement, the mean maximum speed, the highest maximum speed, the maximum acceleration of movement, and the highest maximum acceleration, must be kept in the smart toy because they were in the original design [4].

Finally, accuracy accounts for 20.61% of variance and is a predictor of performance, so sensors that collect data to measure the number of movements, the lowest maximum speed, and the lowest maximum acceleration should also be kept in the smart toy because they were in the original design [4].

Chronological age is not a direct indicator of the level of psychomotor development, although it is related to it. According to the results obtained in the regression analysis, the only factor that predicts children’s chronological age is accuracy. This result agrees with those of a study on dysgraphia [19] in which it was concluded that poor writers were less accurate.

In this research, we have detected at least three factors of interest to describe the level of psychomotor development of children: trembling, speed, and accuracy. This will be the starting point for further research that will focus on exploring the relationships of these factors to a set of motor behaviors of children in their natural settings, school, home, and playground [19,20].

The system is not designed to predict the age of the children, but it was of theoretical interest to know how age is related to the other variables observed. However, it is relevant to know that the variable accuracy, as described in this research, is related to the level of psychomotor development of children and is one of the aspects to be observed in predicting possible difficulties in school, such as dysgraphia [19].

The EDUCERE DDSS could benefit from larger sample sizes to “learn” to detect and classify delays of psychomotor development. Therefore, the following research work for the smart toy should be tested with more toddlers, with and without developmental delays, diagnosed or not. Furthermore, the data obtained from these new experiments will be used for a further validation of the results of the analysis presented in this paper because this new set of data could guarantee that the predictive power of the algorithm stays the same when separating training and test data.

Consequently, the next phase of the research will focus on the objective to establish the criteria for classification of psychomotor development delays and to describe the actions to be performed in each case, relying on the previously validated smart toys. These criteria do not correspond exactly to the conventional diagnostic criteria because the interest of this investigation is to detect slight delays that are usually unnoticed in the standardized tests, which is the added value of this research.

The final mission of the EDUCERE DDSS is starting to be achieved by providing parents, educators, psychologists, and pediatricians with accurate data about potential delays detected. Results obtained initially triggered some advisement to follow-up in some cases leading to messages such as, “the movements and child´s interaction are OK” or “let the child keep playing but visit the specialist in 3 months.”

## Appendix

Multimedia Appendix 1 Descriptions relevant to the experimental procedure.

## Abbreviations

DDSS: developmental delay screening system
EDUCERE: Ecosistema de Detección Ubicua, atenCión y Estimulación tempRana para niños con trastornos del dEsarrollo [Ubiquitous Detection Ecosystem to Care and Early Stimulation for Children with Developmental Disorders]
ICC: intraclass correlation coefficient
IoT: Internet of things
PCB: printed circuit board
