# Supplementary material for: A Smart Toy to Enhance the Decision-Making Process at Children’s Psychomotor Delay Screenings: A Pilot Study
Source: J Med Internet Res. 2017 May 19;19(5):e171. doi: 10.2196/jmir.7533 (PMC5457531; doi:10.2196/jmir.7533)
Supplement: Multimedia Appendix 1 [file jmir_v19i5e171_app1.pdf]

S1: Summary of interactions and phrases used during the experiment

Event	Experimenter	Child
The experimenter make the tower with the stackable cubes in the marked position	"Look, I'm going to make a tower with these cubes right here."	looks at the tower
The experimenter returns the cubes to initial position		
	"Now I would like you to make a tower just like I did"	listens to the experimenter
Cubes send data to collector module	Wait for the child to make the tower (If the child drops the cubes, the experimenter suggests to put them back in the tower)	tries to make the tower
End of the experiment for each child	Thanks child's participation with a verbal reward: "you have done it very well", and with an attractive sticker	receives a sticker as a reward

S2: Criteria for experts' assessment

Numerical score	Meaning
10	Tower well aligned, in the indicated place, performed quickly
9	Tower well aligned, in the indicated place, performed at medium speed
8	Tower well aligned, in the indicated place, performed at slow speed
7	Tower well aligned, in a wrong place, performed quickly
6	Leaning but not falling, made in the indicated place, performed at slow speed
5	Leaning but not falling, made in a wrong place, performed at slow speed
4	The child is able to stack 4 cubes in the indicated place
3	The child is able to stack 3 cubes in the indicated place
2	The child is able to stack 2 cubes in the indicated place
1	The child is able to stack 1 cubes in the indicated place

S3: Professional profile of the experts

Expert	Professional Profile
Developmental Psychologist	Expert in cognitive and language development
Physiotherapist	Expert in therapy for childhood disabilities
Educator 1	Teacher expert in early attention
Educator 2	Expert in teaching for inclusion
